# Erythropoietin Stimulates GABAergic Maturation in the Mouse Hippocampus

**DOI:** 10.1523/ENEURO.0006-21.2021

**Published:** 2021-02-11

**Authors:** Kasifa Khalid, Julia Frei, Mostafa A. Aboouf, Christina Koester-Hegmann, Max Gassmann, Jean-Marc Fritschy, Edith M. Schneider Gasser

**Affiliations:** 1 Institute of Pharmacology and Toxicology, Neuroprotection Group, University of Zurich, Zurich 8057, Switzerland; 2 Institute of Veterinary Physiology, Vetsuisse Faculty, and Zurich Center for Integrative Human Physiology, University of Zurich, Zurich 8057, Switzerland; 3 Neuroscience Centre Zurich, University of Zurich and Eidgenössische Technische Hochschule Zurich, Zurich 8057, Switzerland

**Keywords:** EPOR, inhibition, interneurons, parvalbumin, perineuronal nets, postnatal development

## Abstract

Several neurodevelopmental disabilities are strongly associated with alterations in GABAergic transmission, and therapies to stimulate its normal development are lacking. Erythropoietin (EPO) is clinically used in neonatology to mitigate acute brain injury, and to stimulate neuronal maturation. Yet it remains unclear whether EPO can stimulate maturation of the GABAergic system. Here, with the use of a transgenic mouse line that constitutively overexpresses neuronal EPO (Tg21), we show that EPO stimulates postnatal GABAergic maturation in the hippocampus. We show an increase in hippocampal GABA-immunoreactive neurons, and postnatal elevation of interneurons expressing parvalbumin (PV), somatostatin (SST), and neuropeptide Y (NPY). Analysis of perineuronal net (PNN) formation and innervation of glutamatergic terminals onto PV+ cells, shows to be enhanced early in postnatal development. Additionally, an increase in GABA_A_ergic synapse density and IPSCs in CA1 pyramidal cells from Tg21 mice is observed. Detection of EPO receptor (EPOR) mRNA was observed to be restricted to glutamatergic pyramidal cells and increased in Tg21 mice at postnatal day (P)7, along with reduced apoptosis. Our findings show that EPO can stimulate postnatal GABAergic maturation in the hippocampus, by increasing neuronal survival, modulating critical plasticity periods, and increasing synaptic transmission. Our data supports EPO’s clinical use to balance GABAergic dysfunction.

## Visual Abstract

**Figure:**
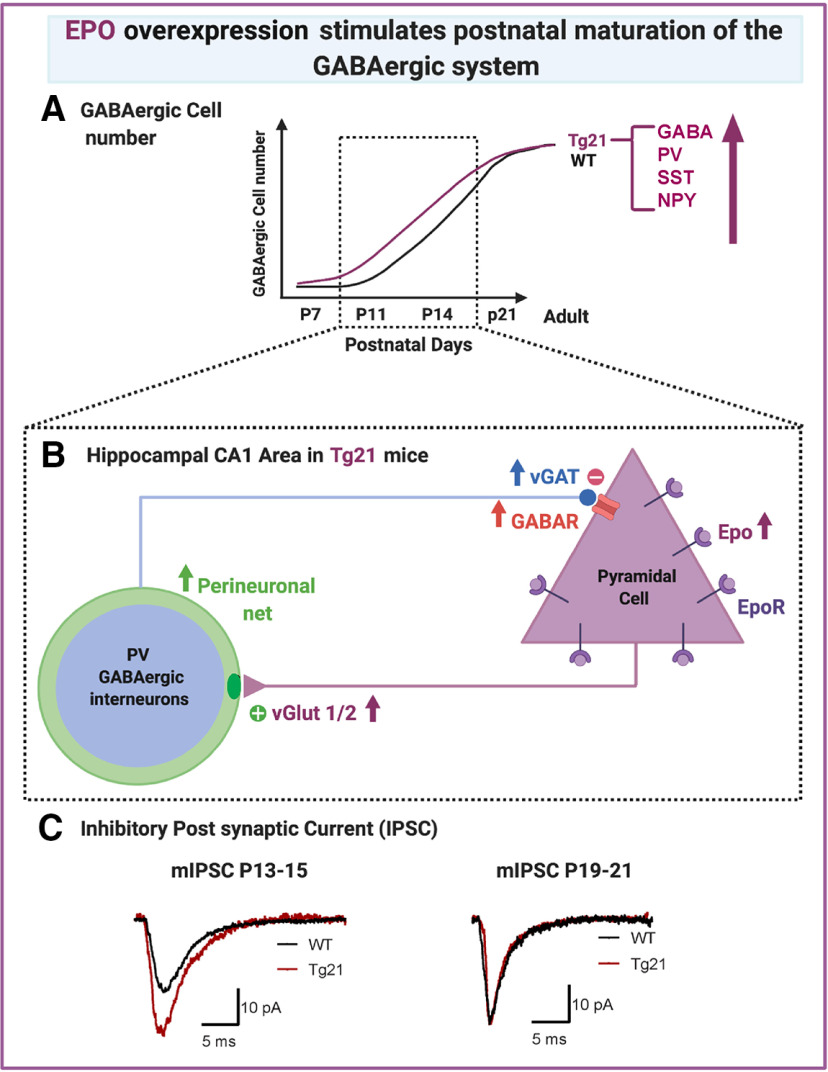

## Significance Statement

Using a mouse model that overexpresses recombinant human erythropoietin (EPO) in the CNS, we observed stimulation of the postnatal maturation of GABAergic transmission in the hippocampus, notably accelerated maturation of parvalbumin (PV)+ interneurons, enhanced glutamatergic inputs onto these interneurons, and enhanced IPSCs onto pyramidal cells. We show that EPO receptors (EPORs) are expressed on pyramidal cells, therefore the impact of EPO on GABAergic maturation is likely to be indirect. Our data show that EPO can modulate hippocampal network maturation and support ongoing trials of the use of EPO in clinical neonatology to stimulate neuronal maturation after perinatal brain injury (PBI).

## Introduction

Perinatal brain injury (PBI) might lead to psychiatric disorders associated with alterations in GABAergic transmission (Marin, 2012; Cunha-Rodrigues et al., 2018; Lacaille et al., 2019). A significant reduction of several markers of GABAergic transmission, including glutamic acid decarboxylase (GAD), GABA, GABA_A_ receptors (GABA_A_Rs), and perturbed parvalbumin (PV) and somatostatin (SST) expression in cortical interneurons, have been reported in the neonatal brain after injury (Robinson et al., 2006; Komitova et al., 2013). In addition, postmortem samples from human preterm infants with brain injury, as well as the hippocampus of rat models of prematurity, showed reduced potassium-chloride co-transporter 2 (KCC2) expression (Jantzie et al., 2014). Perturbations of the GABAergic system in PBI might disrupt the excitatory/inhibitory balance and lead to long-lasting deficits in brain function (Komitova et al., 2013). Therefore, there is an urgent need for new therapeutic strategies protecting the GABAergic system in clinical neonatology.

Erythropoietin (EPO), the erythropoietic hormone (Farrell and Lee, 2004). is a leading therapy in neonatology as a neuroprotective agent (Juul et al., 2015; Juul and Pet, 2015; Natalucci et al., 2016). EPO signaling, leads to activation of several downstream pathways including the STAT5, ERK1/2, and PI3K/Akt pathways (Lombardero et al., 2011). EPO’s immediate neuroprotective effects are anti-apoptotic, anti-inflammatory, and anti-oxidative (Noguchi et al., 2007; Rangarajan and Juul, 2014). In the long-term, EPO stimulates angiogenesis (Zhu et al., 2014), neurogenesis (Castaneda-Arellano et al., 2014). and oligodendrogenesis (Jantzie et al., 2013; Juul et al., 2015). EPO has also been shown to restore deficits in KCC2 expression (Jantzie et al., 2014), and enhance synaptic plasticity and cognition (Adamcio et al., 2008; Kamal et al., 2011; Sargin et al., 2011; Almaguer-Melian et al., 2015), while facilitating inhibitory synaptic transmission (Wojtowicz and Mozrzymas, 2008; Roseti et al., 2020). Nevertheless, it is not yet established whether EPO promotes the development of GABAergic neurons and GABAergic neurotransmission.

EPO and its receptor [EPO receptor (EPOR)] are expressed in human and mouse brain (Digicaylioglu et al., 1995; Marti et al., 1996). Specifically, they are reported to be expressed in the embryonic neocortex in areas close to ventricles and deeper layers, regulating radial migration and laminar positioning of granular neurons (Constanthin et al., 2020). Here, we report that EPORs are highly expressed postnatally in the cornu ammonis (CA)1 hippocampus from mice, increasing their expression to reach a zenith toward adulthood. Additionally, we showed in a transgenic mouse line constitutively overexpressing human EPO in the CNS, without hematopoietic changes (Tg21; Wiessner et al., 2001), a strong activation of the AKT pathways in the postnatal CA1 hippocampus (Jacobs, R. A., Aboouf M. A., Laouafa S., Arias-Reyes C., Koester-Hegmann C., Thiersch M., Soliz J., Gassmann M, and Schneider Gasser E.M., Comm biology, unpublished observations). AKT phosphorylation has a strong antiapoptotic action and can increase the number of GABA_A_Rs on the plasma membrane increasing synaptic transmission in neurons (Wang et al., 2003). Therefore, we hypothesize an important role for EPO signaling in postnatal hippocampal GABAergic maturation.

During the second postnatal week, GABAergic transmission in the CA hippocampus switches from excitatory, because of elevated intracellular chloride concentration, to inhibitory at around postnatal days (P)13–P15 (Tyzio et al., 2007), an age that coincides with a peak in synaptogenesis, and the formation of adult neuronal networks (Ben-Ari et al., 2007). GABAergic transmission is essential for establishing critical periods of enhanced synaptic plasticity during development (Hensch and Bilimoria, 2012). Perineuronal nets (PNNs) are specialized extracellular matrix (ECM) structures composed of chondroitin sulfate proteoglycans that are responsible for synaptic stabilization, a process that influences the closing of critical periods of plasticity. In the hippocampus PNNs are found around the somata and proximal dendrites of PV+ interneurons, with an onset at P8, a time point when the expression of PV increases and also the midpoint in the switch from excitatory to inhibitory GABAergic transmission. PNNs are good indicators of brain maturation and contribute to the maintenance of the excitatory/inhibitory balance (Hensch, 2005; Fowke et al., 2018). Several secreted factors, including neurotrophic factors, also modulate the maturation of inhibitory circuits and consequently the timing of PNN formation (Huang et al., 1999; Begum and Sng, 2017). It remains to be elucidated whether EPO regulates the formation of PNNs.

In this work, we quantified the numbers of interneurons immunoreactive for GABA, PV, SST, and neuropeptide Y (NPY), analyzed the formation of PNNs, evaluated the glutamatergic innervation of PV+ neurons, we quantified the density of GABAergic synapses in the CA1 area and recorded spontaneous IPSC (sIPSC) and miniature IPSC (mIPSC) in pyramidal cells using whole-cell electrophysiology. Finally, we determined, the cellular mRNA expression of EPORs in the CA1 area and evaluated EPORs and PV+ cell numbers in GAD65-cre^Tg/+^, EPOR^fx/fx^ mice. Our data show that EPO increases postnatal neuronal survival, enhances GABA_A_Rs and conductance onto pyramidal cells; increases glutamatergic inputs onto PV+ cells, and accelerates PNN formation. Thus, EPO is a potential drug to stimulate hippocampal GABAergic maturation without causing any network imbalance.

## Materials and Methods

### Animals

Animal experiments were performed following the ARRIVE guidelines and were approved by the Cantonal Veterinary Office of Zurich, Switzerland. Mice were bred on a C57BL/6 background at the Laboratory Animal Service Centre of the University of Zurich and kept in standard housing conditions with food and water provided *ad libitum.* At least three animals per genotype and age from three different litters were used for each experiment.

#### TgN(PDGFB-EPO)322ZbZ (Tg21)

The Tg21 transgenic mouse line overexpressing EPO in the CNS was generated by pronuclear microinjection of the full-length human EPO cDNA driven by the platelet-derived growth factor (PDGF) B-chain promoter into fertilized oocytes derived from B6C3 hybrid mice (Ruschitzka et al., 2000; Wiessner et al., 2001). The resulting hemizygous offspring was then backcrossed with C57BL/6 mice and bred to homozygosity to generate transgenic TgN(PDGFB-EPO)322ZbZ (Tg21) and wild-type (WT) mouse lines with the same genetic background. Experiments were performed in mice of both sexes, and group sizes are reported with the statistical analyses.

#### GAD65-cre,EPOR^fx/fx^

Heterozygous GAD65-Cre (kind gift from H. U. Zeilhofer, University of Zurich, Switzerland) male mice were bred with homozygous EPOR floxed (fx) female mice (kind gift from C. Grimm, University of Zurich, Switzerland). Cre-positive, EPOR^fx/+^ offspring were bred again with homozygous EPOR floxed mice. Cre-positive, EPOR^fx/fx^ offspring was then used for immunostaining and fISH analysis and compared with Cre-negative, EPOR^fx/fx^ mice.

### Immunohistochemistry

#### Tissue preparation for immunoperoxidase staining

WT and Tg21 mice of both sexes were collected at P7, P11, P14, P21, and P60; GAD65-cre-positive, EPOR^fx/fx^ and GAD65-cre-negative, EPOR^fx/fx^ were collected at P11, and anesthetized by intraperitoneal pentobarbital injection (Nembutal: 50 mg/kg; i.p., Kantonsapotheke Zürich). They were then perfused transcardially with PBS (pH 7.4) to rinse blood, followed by fixative containing 4% paraformaldehyde in 0.15 m Na-phosphate buffer, pH 7.4. Brains were immediately dissected, cut sagittal through the midline, and postfixed in the same fixative for 24 h for P7, 18 h for P11, 12 h for P14, and 3 h for P21 and P60 tissue. After postfixation, brains were transferred to 30% sucrose (in PBS) at 4°C for 24–72 h until tissue sank, for cryoprotection. Brains were cut into sagittal serial sections at 50 μm for P7, and 40-μm thickness for other ages using a sliding blade freezing microtome (HM400; Microm). Six serial sections were collected for brains at P7, eight serial sections for P11, 10 serial sections for P14, and 12 serial sections for P21 and P60. Sections were stored at −20°C in antifreeze solution until use.

#### Tissue preparation for immunofluorescence

WT and Tg21 mice of both sexes collected at P11, P14, P21, and P60, were deeply anesthetized with an intraperitoneal injection of sodium pentobarbital (50 mg/kg), and perfused transcardially at a constant flow rate with 8–12 ml/min of ice-cold oxygenated artificial CSF (ACSF), pH 7.4. Following immediate decapitation, the brain was dissected, cut sagittal through the midline and postfixed in ice-cold 4% paraformaldehyde (dissolved in 0.15 m Na-phosphate buffer, pH 7.4) 3 h for P11 and P14 and 90 min for P21 and P60. Brains were then rinsed twice with cold PBS, transferred to 30% sucrose in PBS for cryopreservation and stored for 3 d at 4°C. Brains were frozen with dry ice and kept at −80°C until further use. Sagittal sections were cut as described above.

#### Immunoperoxidase staining

PV-immunoreactive, SST-immunoreactive, NPY-immunoreactive, and CB-immunoreactive interneurons in the hippocampus were quantified in sections processed for immunoperoxidase staining. Free-floating sections were washed three times for 10 min each with Tris-Triton buffer, pH 7.4 followed by incubation with rabbit primary antibody against PV (Table 1) in a solution containing 2% Triton X-100 and 2% normal goat serum (NGS) in Tris-Triton buffer, pH 7.4, overnight at 4°C under continuous agitation. The next day, the sections were rinsed again three times for 10 min each with Tris-Triton buffer, pH 7.4 before being incubated at room temperature (RT) for 30 min with the biotinylated secondary antibody (goat anti-rabbit, Jackson ImmunoResearch, 1:300) in a solution containing 2% NGS in Tris-Triton buffer, pH 7.4. After another washing step of three times 10 min, the sections were incubated for 30 min in avidin-biotin complex solution (Vectastain Elite kit; Vector Laboratories) and rinsed again three times for 10 min. To allow equal penetration of the tissue, the sections were preincubated in diaminobenzidine (DAB) solution (0.5 × *g*/L DAB in Tris-Triton buffer, pH 7.7) for 5 min under agitation, before the reaction was started by adding 2 ml of DAB solution containing 0.01% hydrogen peroxidase. After 5–7 min, depending on the intensity of the staining, the reaction was stopped by transferring the sections into ice-cold PBS followed immediately by another washing step of three times 10 min in PBS. The sections were mounted on gelatin-coated glass slides and left to dry overnight. On the following day, they were dehydrated in ethanol of increasing concentrations (2 × 70%, 2 × 96%, 3 × 100%) for 5 min each followed by clearing in xylene four times for 5 min. Finally, the sections were cover-slipped with Eukitt mounting medium (Merck).

**Table 1:** Primary antibodies used for immunohistochemistry

Target	Host species	Dilution	Catalog #	Company/origin	References
γ-aminobutyric acid (GABA)	Rabbit	1:1000	A-2052	Sigma	Dzyubenko et al. (2017)
Neuronal nuclei (NeuN)	Mouse monoclonal	1:1000	MAB377	Merck	Mullen et al. (1992)
PV	Rabbit	1:5000	PV-28	SWant	Vaghi et al. (2014)
NPY	Rabbit	1:1000	T-4069	Peninsula Lab.	Mackay al. (2019)
SST	Rabbit	1:500	sc-13099	Santa Cruz	Yang et al. (2008)
VGAT	Mouse monoclonal	1:2000	131011	Synaptic Systems	Pan-Vazquez et al. (2020)
Gephyrin	Rabbit monoclonal	1:1000	147008	Synaptic Systems	Schneider Gasser et al. (2006)
GABA_A_ R γ_2_	Guinea pig	1:2000	N/A	Home made	Fritschy and Mohler (1995)
vGluT1	Guinea pig	1:1000	135304	Synaptic Systems	Wei et al. (2016)
vGluT2	Guinea pig	1:3000	AB2251	Merck	Holló et al. (2017)
Cleaved caspase-3	Rabbit	1:200	9661	Cell Signaling	Teoh et al. (2017)

#### Immunofluorescence staining

Double or triple immunofluorescence staining was used to analyze multiple markers within the same section. Free-floating sections were washed three times for 10 min each in Tris-Triton buffer, pH 7.4 before being incubated overnight at 4°C under continuous agitation with the primary antibodies raised in different species (Table 1) in a solution containing 2% Triton X-100 and 2% NGS in Tris-Triton buffer, pH 7.4. The next day, the sections were again rinsed three times for 10 min with Tris-Triton buffer, pH 7.4 followed by incubation with secondary antibodies raised in goat against the different species of the primary antibodies and coupled to either Alexa Fluor 488, Cy3, or Alexa Fluor 647 [or the plant lectin *Wisteria floribunda* agglutinin (WFA) coupled to Cy3 for staining of PNNs] in a solution containing 0.5 μl DAPI and 2% NGS in Tris-Triton buffer, pH 7.4 at RT for 30 min in the dark. After another washing step of three times 10 min with Tris-Triton buffer, pH 7.4, sections were mounted on gelatin-coated glass slides and cover-slipped with Dako fluorescence mounting medium (Dako).

### Fluorescence *in situ* hybridization (fISH)

#### Tissue preparation for fISH

WT, Tg21 mice of both sexes at postnatal ages P7, P11, P21, and P60, and GAD65-cre-positive, EPOR^fx/fx^, and GAD65-cre-negative, EPOR^fx/fx^ mice at postnatal age P11, were deeply anesthetized with an intraperitoneal injection of sodium pentobarbital (50 mg/kg) followed by decapitation and dissection of brain tissue on ice. Hemispheres where then frozen on dry ice and stored at −80°C until use. Serial brain sections of 10 μm were cut using a cryostat (Leica Biosystems), mounted on Superfrost slides and stored at −80°C.

#### fISH

fISH of murine EPOR (RNAscope Probe-Mm-Epor) was performed, using the RNAscope Multiplex Fluorescent reagent kit v2 from Advanced Cell Diagnostics and the fluorophore Opal 520 from PerkinElmer. Positive and negative control probes (RNAscope 3-plex positive and negative control probes) were always run in parallel.

Fresh frozen tissue was postfixed for 30 min at 4°C in 4% paraformaldehyde prepared in 0.15 m Na-phosphate buffer (pH 7.4), treated 10 min with hydrogen peroxide at RT, target retrieval for 10 min at 85°C (RNAscope Target Retrieval reagent) and protease treatment with Protease Plus for 30 min at 40°C. Probes were hybridized for 2 h at 40°C. Slides were washed in Tris-Triton buffer, pH 7.4 followed by amplification steps (RNAscope Amp1) and signal development (HRP-C1 + fluorophore 1). Finally, slides were incubated for 3 min in DAPI and coverslipped.

### Image acquisition

#### Bright-field microscopy

Sections processed for immunoperoxidase staining were visualized and photographed with an Axioscope 2 microscope (Carl Zeiss AG) equipped with a color digital camera (AxioCam MRc5) and its corresponding software, AxioVision 4.5 (Carl Zeiss AG). Images of the whole hippocampus were taken with bright-field illumination using a 5× objective (NA 0.15).

#### Fluorescence microscopy

Images of cleaved caspase**-**3 and DAPI samples were imaged with a Zeiss Axio Imager 2 fluorescent microscope (Carl Zeiss AG). Image stacks (five slices, 2-μm intervals) from CA3 and CA1 areas were taken with a 10× objective, with a scan zoom of 1× and image size of 1024 × 1024 pixels.

#### Confocal laser-scanning microscopy

Immunofluorescence GABA and NeuN-stained tissue sections were imaged using a Zeiss LSM 700 confocal laser scanning microscope (Carl Zeiss AG) with a 40× oil immersion objective with a numerical aperture (NA) of 1.4. Images were taken as *z*-stacks (five slices, 1-μm intervals) with a scan zoom of 1× for CA3 area and 0.5× for the CA1 area and an image size of 1024 × 1024 pixels.

For GABAergic synaptic cluster analysis, vesicular GABA transporter (VGAT), gephyrin and GABA_A_Rγ2 subunits were stained, and sections were imaged using a Zeiss LSM 800 confocal laser scanning microscope (Carl Zeiss AG). For glutamatergic inputs, vesicular glutamate transporter (vglut)1-2 and PV cells stained sections were imaged. A 63× oil immersed objective with a NA of 1.4 was used for synapse analysis. Images were taken as *z*-stacks (five slices, 0.2-μm intervals) with a scan zoom of 1.5× and an image size of 1024 × 1024 pixels. Imaging parameters were kept constant over all conditions. After acquisition, images were processed using 2D super resolution Airy Scan processing run in automated mode. image acquisition was conducted in the stratum pyramidale and stratum radiatum of the CA1 and CA3 region.

For analysis of PNNs around PV+ interneurons, double-stained sections were imaged using a Zeiss LSM 800 confocal laser scanning microscope (Carl Zeiss AG) with a 10× objective (NA 0.45) and taking image stacks composed of 22 slices at a *z*-intervals of 0.4 μm. Image acquisition was performed in the CA1 and the CA3 area of the hippocampus and image settings were kept constant between genotypes within every age.

##### fISH image acquisition

Samples were imaged with a Zeiss LSM 700 confocal laser scanning microscope (Carl Zeiss AG). Image stacks (six optical sections, 0.5-μm step size) from CA1 and CA3 stratum pyramidale and radiatum were acquired with at 40× objective, N.A. 1.4.

#### Stereology

Unbiased counting of PV+, SST+, and NPY+ interneurons in the hippocampus was performed using the 10× objective (NA 0.45) of an Axioplan 2 bright-field microscope (Carl Zeiss AG) equipped with a digital camera (MicroFIRE, Optronics AG). First, the hippocampal CA1 and CA3 areas containing the different subregions: stratum oriens, stratum pyramidale, stratum radiatum and stratum lacunosum moleculare, were delineated with Mercator Pro software (Explora Nova) according to the mouse brain atlas (Paxinos, 2007). Subsequently, immunoreactive cells were counted in each of these areas with no distinction of subregions. Data collection was done in serial sections throughout the whole hippocampus, analyzed with a serial sampling fraction (ssf) of three for P7, four for P11, five for P14, and six for P21 and P60. Six animals per genotype and age were analyzed, except for SST analysis where only four animals per genotype were analyzed.

The total volumes (V_tot_) of the CA1 and CA3 were calculated from the ssf, the areas delineated in every section (A_i_ – A_n_; *n* = number of sections analyzed) and the section thickness (h) as follows:

$$V_{tot}=ssf\times \overset{n}{\underset{i=1}{\sum }}A_{i}\times h.$$

Subsequently, the total number of immuno-positive cells (Q_tot_) in the CA1 and CA3 were calculated using the ssf and the positive cell numbers per section (Q_i_):

$$Q_{tot}=ssf\times \overset{n}{\underset{i=1}{\sum }}Q_{i}.$$

Total number of NeuN+, and GABA+ cells were quantified with the optical Fractionator using the Stereo Investigator software (v10.50, MBF Bioscience) equipped for fluorescence imaging, with a 63× lens (Zeiss 1.4 Oil). Neu+ and GABA+ cells were counted independently in a frame of 40 × 40 μm with a step size of 120 μm. Total cell numbers (*N*) were calculated using the formula: $N=\sum Q.\left(\frac{1}{asf}\right)\left(\frac{1}{ssf}\right)$, where ∑Q is the total number of counted cells, and asf the area sampling factor. A mean of 200 cells was counted per animal. For more details on stereological estimates (Slomianka and West, 2005).

### Optical density analysis

PV immunoperoxidase staining intensity was assessed by densitometry analysis using the MCID software (MCID Elite 6.0, InterFocus Imaging Ltd.). Images were digitzed using a precision illuminator (Northern Light Model B95, Imaging Research Inc., Brock University, St. Catharines, Canada) and CoolSNAP cf. photo-camera (Photometrics) with a Micro-Nikkor (55 + 12 mm) objective (Nikon Corp.). Next, a gray value calibration (Kodak step tablet no. 310ST607) was performed, and the intensity was measured in the different regions of interest (ROIs). To correct for variations in background staining, the intensity value was normalized to the intensity of the corpus callosum. A total of five images per animal and three animals per genotype, were analyzed.

### Caspase-3 analysis

Four brain sections were imaged per mouse and maximum intensity projections were created from *z*-stacks. Cell densities of caspase-3+ cells were directly quantified from each field of view. All imaging parameters were kept constant between groups. Images were processed with Fiji ImageJ (NIH).

### PNN analysis

Raw confocal images were preprocessed to 8-bit grayscale tiff files, before being z-projected into a sum slices image using ImageJ (NIH) software, allowing the image to be analyzed in two dimensions without losing any information. In a next step, PV+ cells and PNN number, size and area were analyzed using the Perineuronal net Intensity Program for the Standardization and Quantification of ECM Analysis (PIPSQUEAK) macro (Slaker et al., 2016) in FIJI (ImageJ, NIH) software. The macro was run in semi-automated mode, which allowed the manual confirmation of PV+ cells and PNNs. Background subtraction was achieved with Rolling Ball Radius followed by the selection of 20 ROIs around the perimeter of an image. After removal of high and low outlier ROIs, a mean background value could be calculated to remove variability in background staining (Slaker et al., 2016). Analysis of PV and PNN fluorescence intensity was performed in average projection images from *z*-stacks using a custom-made macro in ImageJ (NIH) software. Processing of both channels included thresholding method Yen for PNN and Otsu for PV, background subtraction, Gaussian blurring, and size restrictions. Six animals per age and genotype were used for the analysis. Data points represent average values of the total quantified cells per animal.

### Synaptic cluster analysis

Morphologic quantification of synaptic cluster densities was performed in maximal intensity projection images from three images in the *z*-stack, using a custom-made macro in ImageJ software (NIH). Processing of all channels included background subtraction with Rolling Ball Radius, Gaussian blurring, thresholding for selecting ROIs of high staining intensity (representing local accumulation of synaptic proteins), as well as shape (circularity: 0.4–1) and size (minimal area 0.1 μm^2^) restrictions for cluster detection, using the same parameters in all images per genotype and age. GABAergic synapses were detected based on colocalization of a γ_2_-GABA_A_R, gephyrin, and VGAT-immunofluorescence. vGlut1-2 inputs onto PV+ cells were quantified by colocalization of both channels. To determine apposition of presynaptic markers (vGAT and vGluT), the size of the cluster was increased by one pixel all around (Tyagarajan et al., 2011; Früh et al., 2016). Four mice per genotype and age were used for the analysis.

#### fISH analysis

Analysis was done in *z*-stack images with maximum intensity projection, using a custom-made cluster analysis macro in ImageJ (NIH) software. Processing of EPOR particles (green channel) was separately analyzed, with background subtraction using rolling ball radius, Gaussian blurring, and thresholding for selecting ROIs of high staining intensity, as well as shape (0.5–1 circularity) and size (0.1–1 μm in diameter) restrictions. The same parameters were used in all images per genotype and age. Six animals were used for the analysis.

### Electrophysiology

#### Acute brain slice preparation

WT and Tg21 in the postnatal age ranges of P13–P15 and P19–P22 were briefly anaesthetized with isoflurane and decapitated. The number of recordings for each group are as follows; at P13–P15, WT *n*_(cells)_ = 8, *n*_(animals)_ = 4 and for Tg21 *n*_(cells)_ = 9, *n*_(animals)_ = 5; at P19–P22, WT *n*_(cells)_ = 8, *n*_(animals)_ = 6 and for Tg21 *n*_(cells)_ = 8, *n*_(animals)_ = 6. The brain was quickly removed and transferred to ice-cold dissection solution containing 65 mm NaCl, 2.5 mm KCl, 1.25 mm NaH_2_PO_4_, 25 mm NaHCO_3_, 7 mm MgCl_2_, 0.5 mm CaCl_2_, 25 mm glucose and 105 mm sucrose, saturated with 95% O_2_ and 5% CO_2_. 350 μm-thick sagittal slices containing the hippocampus were cut from the tissue block with a vibratome (Leica) and kept in oxygenated ACSF (315 mOsm) containing 125 mm NaCl, 2.5 mm KCl, 1.25 mm NaH_2_PO_4_, 25 mm NaHCO_3_, 1 mm MgCl_2_, 2 mm CaCl_2_, and 25 mm glucose at 32°C for 25 min and then at RT until use.

#### Whole-cell recordings

For recording, individual slices were transferred to a recording chamber perfused with oxygenated ACSF (as aforementioned) at a flow rate of 1–2 ml/min at RT (21–23°C). Whole-cell patch clamp recordings were made from hippocampal CA1 pyramidal neurons visualized using differential interference contrast (DIC) with an upright microscope (Axioscope, Examiner.A1, Carl Zeiss) at a low, 10× (water immersion objective) magnification; high, 63× (water immersion objective) magnification was used for approaching the cell and achieving a high-resistance (gigaohm) seal. sIPSCs were recorded from CA1 pyramidal cells, clamped at a holding potential of –70 mV, in the presence of 20 μm NBQX, 50 μm AP-5 and 0.5 μm Strychnine to block glutamatergic and glycinergic transmission. For mIPSCs, 1 μm tetrodotoxin (TTX) was further added to block events caused by action potentials firings. Recordings with an unstable baseline or a holding current greater than −120 pA were rejected. Recording pipettes were made from borosilicate glass (1.5–1.8 O.D., 0.2 mm thick, Kimble), pulled with a vertical puller (Narishige PC-10), had resistances of 7–9 MΩ when immersed in ACSF and filled with internal solution containing the following: 130 mm K-gluconate, 5 mm NaCl, 10 mm HEPES, 1 mm EGTA, 5 mm Mg-ATP, and 0.5 mm Na-GTP; pH 7.4, 290–300 mOsm. sIPSCs and mIPSCs were recorded with an internal solution containing the following: 120 mm CsCl, 4 mm MgCl_2_, 10 mm HEPES, 10 mm EGTA, 2 mm Mg-ATP, and 0.5 mm Na-GTP; pH 7.4, 290–310 mOsm. Recordings were performed using Multiclamp 700B amplifier (Molecular Devices), data were digitized with Digidata 1440A (Molecular Devices) and acquired with the acquisition software Clampex 10.0 (Molecular Devices). All experiments were performed at RT (21–23°C).

sIPSCs and mIPSCs currents were filtered off-line using a Butterworth low-pass filter (2 kHz), digitized at 10 kHz and analyzed as 2-min epochs following the addition of the pharmacological blockers for 2 min, using the Mini-Analysis Program 6.0.7 (Synaptosoft,). Recordings with leak increasing >120 pA and access resistance changing >30% between the beginning and the end of the recording were discarded. At least 200 events were analyzed for any condition in all experiments. Synaptic events were identified by setting the event detection threshold at least 2-fold higher than the baseline noise level and by ensuring that events had (1) rise times faster than the decay time, (2) rise times >0.5 ms, and (3) decay times >1.5 ms; only events adhering to these parameters were included in further analysis. Event amplitudes, interevent intervals, rise and decay times were averaged within each experiment. The frequencies were calculated from the interevent intervals and the resulting means were averaged between experiments.

### Statistical analyses

All statistical analyses were performed using GraphPad Prism 8 (GraphPad Software). Parametric data with two conditions were analyzed using an unpaired, two-tailed Student’s *t* test. Data in different brain areas that were influenced by one factor were analyzed with multiple *t* test. To compare data influenced by two factors, a two-way ANOVA with Bonferroni multiple comparisons *post hoc* test was used. Probability distributions were tested using the Kolmogorov–Smirnov (KS) test. For all statistical tests, *p* < 0.05 was considered statistically significant. Data are presented as mean ± SD. In order to confirm appropriate sample sizes for experiments, a Power Analysis was conducted using G*Power software (Heinrich Heine Universität, Düsseldorf, Germany; Faul et al., 2007). Changes in total PV number across ages was evaluated in R program with a square root curve polyfit (yp = sqrt” number + age + genotype) and the interference value was evaluated with grofit.

## Results

### Increased number of GABA-immunoreactive neurons in the hippocampus from Tg21 mice

**Figure 1.**
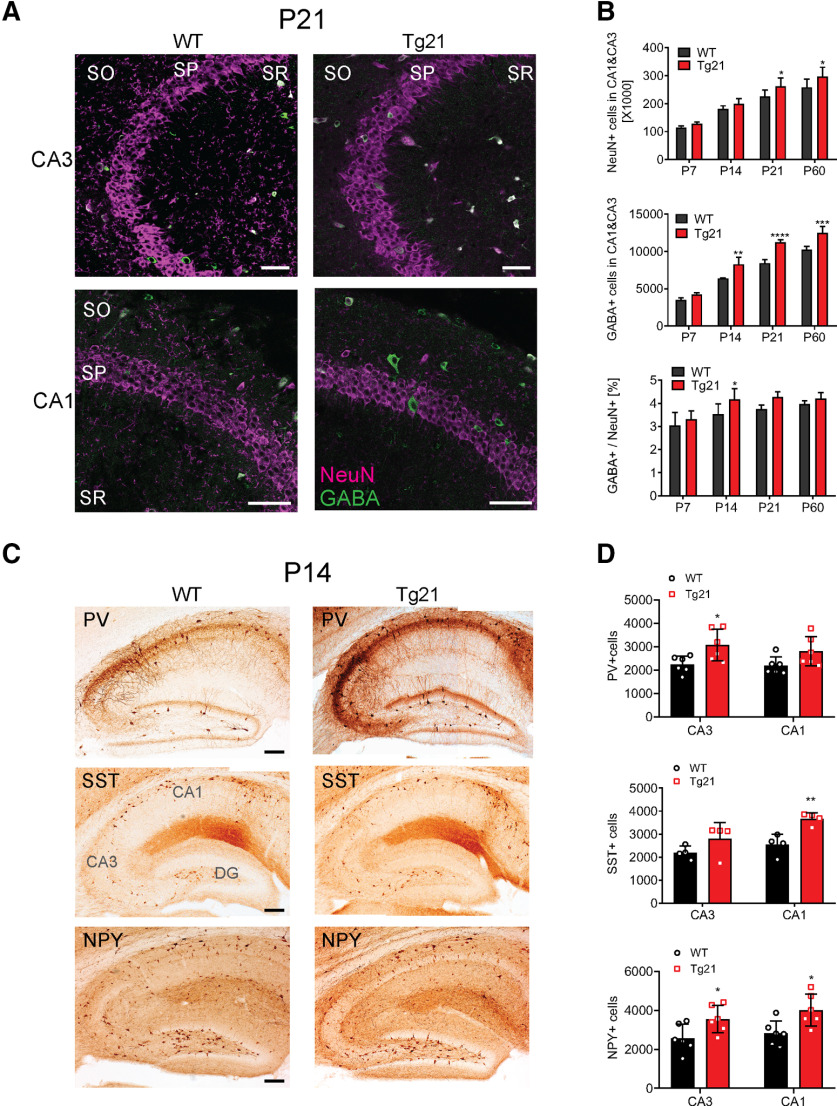
Increased number of GABA-immunoreactive neurons in the hippocampus from Tg21 mice. ***A***, Representative images of double immunofluorescence staining against GABA (green) and NeuN (magenta) for CA3 and CA1 areas of WT and Tg21 mice at P21. so: stratum oriens, sp: stratum pyramidale, sr: stratum radiatum. ***B***, Unbiased quantification of NeuN+ (upper graph) and GABA+ (middle graph) cells in total CA3 and CA1 area of WT and Tg21 mice, showing age-specific differences between genotypes. Ratio of GABA+/NeuN+ cells (lower graph) in WT and Tg21 mice, shows a significant increase of GABA+ cells at P14. Data are given as mean ± SD of total neuronal numbers in the CA1 and CA3 area of the hippocampus; *N* = 6 animals per age and genotype. Two-way ANOVA test, *p <* *0.05, **p <* *0.01, ***p <* *0.001, ****p < 0.0001. Scale bar: 50 μm. ***C***, Representative images of PV+, SST+, and NPY+ immunoperoxidase staining in hippocampus of WT and Tg21 mice at P14 illustrate the stronger immunoreactivity in PV, SST, and NPY in Tg21 mice. DG: dentate gyrus. ***D***, Unbiased quantification of the total cell numbers in CA3 and CA1 areas, revealing increased numbers of PV+ cell in CA3, increased number of SST+ cells in CA1, and increased number of NPY+ cells in CA3 and CA1 in Tg21 mice. Data are given as mean ± SD, *N* = 6 animals per genotype for PV and NPY and *N* = 4 animals per genotype for SST staining. Multiple *t* test; *p <* *0.05, **p <* *0.01. Scale bar: 200 μm.

To determine whether constitutive overexpression of human EPO in the CNS influences GABAergic neurotransmission in the hippocampus, the total number of neurons (immunoreactive for NeuN) and the total number of GABA-immunoreactive neurons were stereologically quantified together in the CA3 and CA1 regions of Tg21 mice at P7, P14, P21, and P60 and compared with the WT control mice (Fig. 1*A*,*B*). Representative images of the CA3 and CA1 areas double-stained for NeuN and GABA at P21 are shown in Figure 1*A*. A significant effect of age and genotype was observed for NeuN-positive cells in the CA area, with increased numbers in Tg21 mice at P21 and P60 (two-way ANOVA, *F*_(1,40)_ = 16.3, *p *=* *0.0002; Fig. 1*B*, upper graph). Likewise, the number of GABAergic neurons was higher in the Tg21 mice starting at P14 (two-way ANOVA, *F*_(1,40)_ = 77.90, *p *<* *0.0001; Fig. 1*B*, middle graph). The early increase of GABA immunoreactivity resulted in a significantly higher ratio of GABA cells in Tg21 mice at P14 (two-way ANOVA, *F*_(1,40)_ = 16.53, *p *=* *0.0002; Fig. 1*B*, lower graph).

To determine whether this early increase of GABA immunoreactivity at P14 occurs in specific subpopulations of interneurons, we quantified the total number of PV+, SST+, and NPY+, interneurons in CA1 and CA3 at this age (Fig. 1*C*,*D*). The results showed region-specific differences, being the increased number of GABAergic neurons in CA3 mainly because of an increase in PV+ and NPY+ cells (multiple *t* test, *p *=* *0.024 and *p *=* *0.044, respectively; Fig. 1*D*), whereas in CA1 mainly because of SST+ and NPY+ cells (multiple *t* test, *p *=* *0.005 and *p *=* *0.018, respectively; Fig. 1*D*). These findings indicate that EPO overexpression accelerates maturation of interneuron subpopulations in a region-specific manner during postnatal development.

### Early onset of PV expression in the hippocampus from Tg21 mice

**Figure 2.**
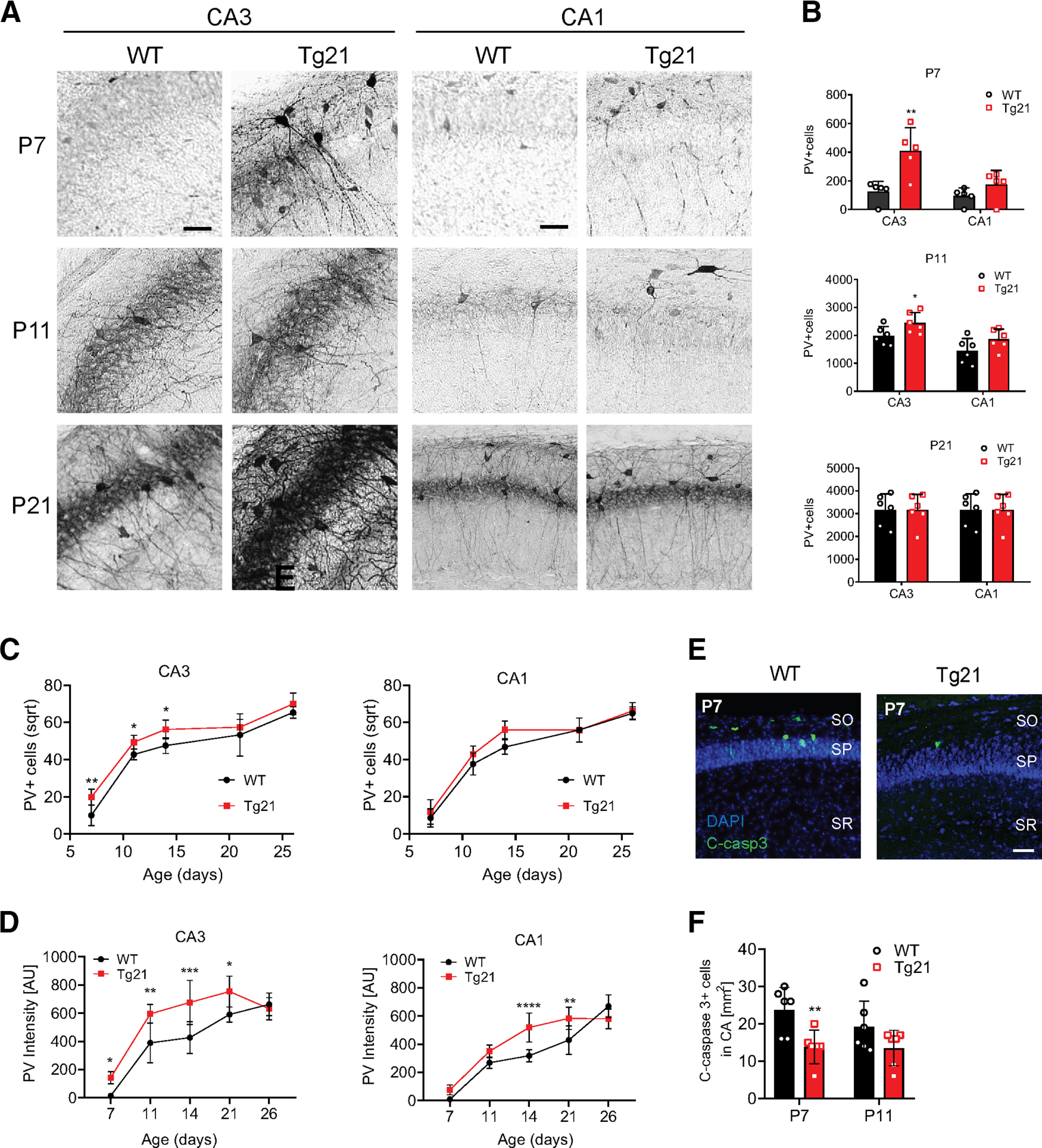
Early onset of PV expression in the hippocampus from Tg21 mice. ***A***, Representative images of PV immunoperoxidase staining in CA3 and CA1 areas of WT and Tg21 mice at P7, P11, and P21. Note the earlier appearance of PV+ cells in the CA3 area (P7). Scale bar: 50 μm. ***B***, Unbiased quantification of the total PV+ cell numbers at each represented postnatal time point for WT and Tg21 mice. Multiple *t* test; *p <* *0.05, **p <* *0.01. ***C***, Significant differences in PV+ cell numbers between genotypes occur in CA3 area at P7, P11, and P14. Grofit interference analysis, *p <* *0.05, **p <* *0.01. ***D***, Significant difference in PV+ intensity is to observe in the CA3 area at postnatal ages: 7, 11, 14, and 21; and in the CA1 area at postnatal ages: 14 and 21. Data are given as scatter dot plots and mean ± SD, *N* = 6 animals per genotype. Two-way ANOVA test; *p <* *0.05, **p <* *0.01, ***p <* *0.001, ****p <* *0.0001. ***E***, Representative images of cleaved caspase-3 (green) and DAPI (blue) staining in CA1 areas of WT and Tg21 mice at P7. Scale bar: 50 μm. ***F***, Cleaved caspase-3 quantification in WT and Tg21in CA3 and CA1 area at P7 and P11. A significant reduction in apoptosis at P7 is observed in Tg21 mice. Two-way ANOVA; *p <* *0.05.

Given the importance of PV+ cells for the regulation of pyramidal cell activity and synchronization during postnatal development, we further explored the effect of EPO on PV+ cell numbers, focusing on the CA1 and CA3 areas at P7, P11, P14, P21, and P26 (Fig. 2). These time points were selected because PV immunoreactivity in the mouse hippocampus appears between P4–P7 and peaks between P14 and P21 (Solbach and Celio, 1991). Quantification of immunolabeled PV cells revealed a highly significant age effect in CA3 and CA1 regions, along with a genotype effect in CA3, strongest at P7, where PV+ cells were still absent in most WT mice but already expressed in all Tg21 mice (multiple *t* test, *p *=* *0.007; Fig. 2*B*). Also, at P11 PV+ cells were increased in number in CA3 (multiple *t* test, *p *=* *0.046; Fig. 2*B*). Normalization and analysis [Poly fit (yp = sqrt(10), y, 1)] of PV cells across development showed a significant increase in PV+ cells at P7, 11 and 14 in CA3 (grofit analysis, *p *=* *0.0003; Fig. 2*C*). Interestingly, not only was the number of PV+ cells higher in the CA3 area at P7, P11, and P14, but the neuropil was also more intensely stained including at P21, as illustrated in Figure 2*A* and measured by optical density (two-way ANOVA, *F*_(1,50)_ = 32.43, *p *<* *0.0001; Fig. 2*D*). In the CA1 area a higher PV staining intensity was measured at P14 and P21 (two-way ANOVA, *F*_(1,48)_ = 21.29, *p *<* *0.0001; Fig. 2*D*).

Taken together, our results point to an accelerated maturation of GABAergic neurons in the hippocampus, along with a neurotrophic effect resulting in higher numbers of all neurons persisting until reaching adulthood in Tg21 mice.

Because EPO is an antiapoptotic cytokine (Ghezzi and Brines, 2004) and many interneurons undergo programmed apoptosis early in postnatal development (Southwell et al., 2012; Priya et al., 2018), we analyzed apoptosis in the CA1 and CA3 areas at postnatal ages P7 and P11 (Fig. 2*E*,*F*). A reduction in cleaved caspase-3+ cells was observed in the stratum pyramidale and stratum oriens at P7 in Tg21 mice (two-way ANOVA, *F*_(1,20)_ = 11.29, *p *=* *0.003; Fig. 2*E*,*F*). Thus, EPO promotes survival of neurons at the second postnatal week.

### EPO influences PNN formation around PV+ cells in the hippocampal CA1 and CA3 areas

**Figure 3.**
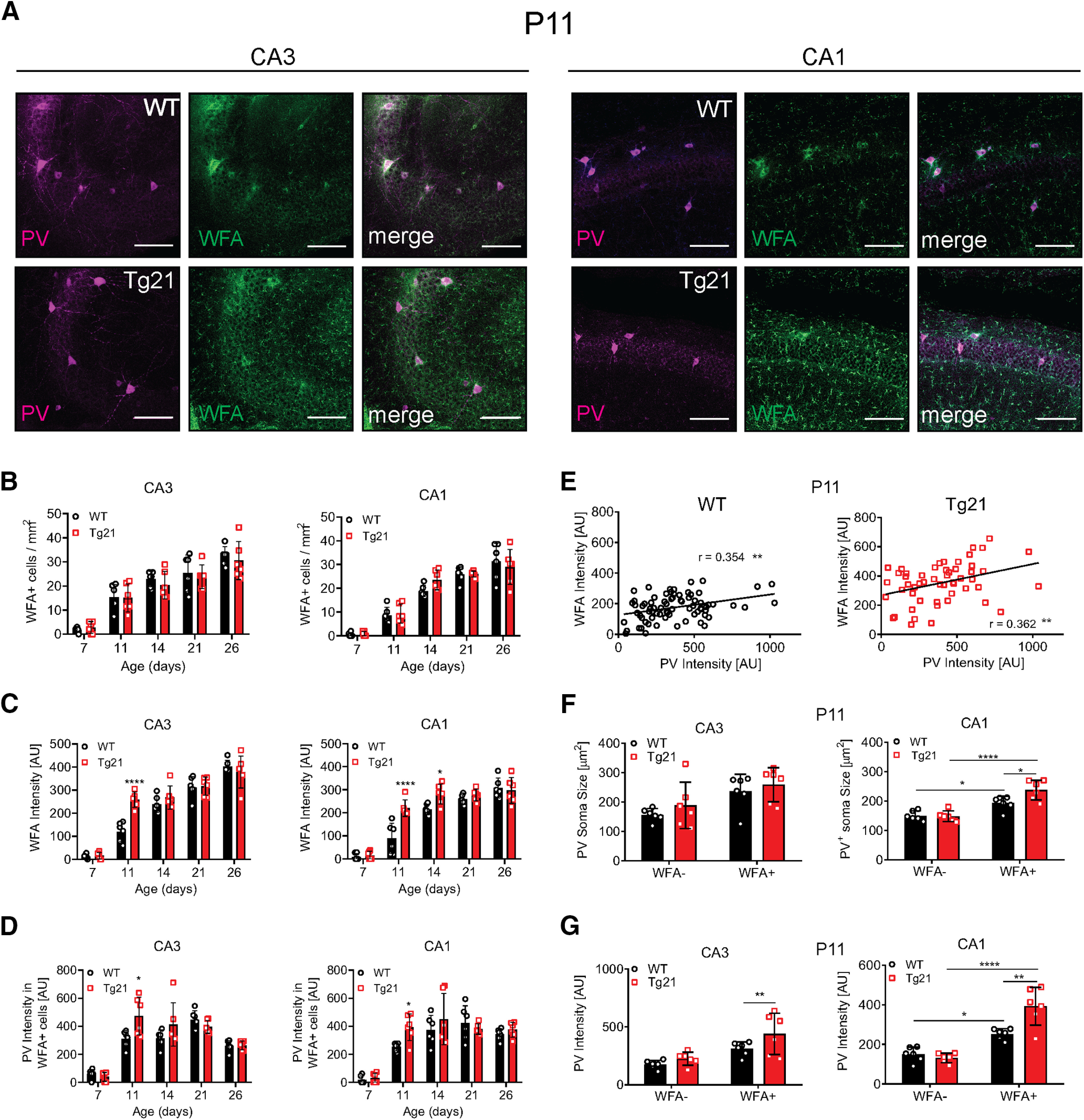
EPO influences PNN formation around PV+ cells in the hippocampal CA1 and CA3 areas. ***A***, Representative images of double labeling for PV immunoreactivity (magenta) and WFA fluorescence that identify PNNs (green) and merged images in CA3 and CA1 areas at P11. Scale bar: 100 μm. ***B***, Quantification of PV+ cells surrounded by PNN (WFA+) in CA3 and CA1 across postnatal ages. No increase in WFA+ cells nor change in the onset is observed between genotypes. ***C***, WFA intensity in CA3 and CA1 across postnatal ages. WFA fluorescence intensity is stronger in Tg21 mice CA3 area at P11 and in CA1 area at P11 and P14. ***D***, PV intensity in WFA+ cells in CA3 and CA1 area across postnatal ages. PV intensity is stronger in CA3 and CA1 areas at P11 in Tg21 mice. ***E***, Correlation analysis of PV and WFA fluorescence intensity in WT and Tg21 mice CA1 area at P11. r: Pearson correlation. ***F***, PV+ cells soma size at P11. PV+ cells covered by WFA have larger cell somas in CA3 and CA1 area from WT and Tg21 mice. WFA+/PV+ cells are larger in Tg21 mice CA1 area. ***G***, PV immunofluorescence intensity at P11. PV intensity is stronger in WFA+ cells, and the intensity is even higher in Tg21 mice in CA3 and CA1 areas. Graphs (***B–F***) show scatter dot plots and mean bars ± SD, *N* = 6 animals per age and genotype. Two-way ANOVA; *p <* *0.05, **p <* *0.01, ***p <* *0.001, ****p <* *0.0001.

To determine whether EPO increases PV+ cell maturation and synapse stabilization, we evaluated the formation of PNNs around PV+ cells, at P7, P11, P14, P21, and P26 in Tg21 and WT mice. The plant lectin WFA, which selectively binds to ECM glycoproteins in the PNNs, was visualized together with PV immunofluorescence (Fig. 3). The following parameters were evaluated in CA1 and CA3 areas: total number of PV+ cells surrounded by PNNs (Fig. 3*B*), WFA fluorescence intensity (Fig. 3*C*), PV fluorescence intensity (Fig. 3*D*), correlation between PV and WFA intensity at P11 (Fig. 3*E*). Additionally, soma size and PV intensity were compared between PV+ cells surrounded or not by PNN at P11 (Fig. 3*F*,*G*). No genotype effect was observed in number of PV+/WFA+ cells (Fig. 3*B*). However, the intensity of WFA was significantly higher in Tg21 mice in CA3 and CA1 areas at P11, and in CA1 at P14 (CA3: two-way ANOVA, *F*_(1,50)_ = 8.71, *p *=* *0.0048 and CA1: two-way ANOVA, *F*_(1,50)_ = 18.36, *p *<* *0.0001; Fig. 3*C*). Also, PV intensity of WFA surrounded cells was higher in CA3 and CA1 at P11 (CA3: two-way ANOVA, *F*_(1,50)_ = 3.8, *p *=* *0.049 and CA1: two-way ANOVA, *F*_(1,50)_ = 3.78, *p *=* *0.049; Fig. 3*D*), which suggest that constitutive EPO overexpression modulates the formation of PNNs at this early stage of development. Since quantification of PV+ cells surrounded by PNNs (i.e., WFA+) showed similar values in the CA3 and CA1 areas, the increased cell numbers at P7, P11 and P14 in CA3 from Tg21 mice reflects uncovered (i.e., WFA–) cells. At P11, a positive correlation between PV and WFA fluorescence intensity was seen in both genotypes [Pearson correlation analysis (*r*), *p *<* *0.01; Fig. 3*E*]. Also the soma size of PV+/WFA+ cells was significantly larger for both genotypes in the CA3 and CA1 areas (CA3, two-way ANOVA, *F*_(1,20)_ = 8, *p *=* *0.01; CA1, two-way ANOVA, *F*_(1,20)_ = 25.26, *p *<* *0.0001; Fig. 3*F*) and even larger in the Tg21 mice than WT in the CA1 area (CA1, two-way ANOVA, *F*_(1,20)_ = 4.893, *p *=* *0.0388; Fig. 3*F*). A comparison of PV fluorescence intensity of PV+/WFA– and PV+/WFA+ cells showed that interneurons surrounded by PNNs have a significantly higher PV staining intensity than PV+/WFA– cells in both genotypes (CA3, two-way ANOVA, *F*_(1,20)_ = 9.814, *p *=* *0.0052; CA1, two-way ANOVA, *F*_(1,20)_ = 21.7, *p *=* *0.0002; Fig. 3*G*), likewise with a significant genotype effect in the CA1 area, with PV+/WFA+ cells in Tg21 mice being more strongly fluorescent than in WT (CA1, two-way ANOVA, *F*_(1,20)_ = 5.342, *p *=* *0.037; Fig. 3*G*). In brief, our data show that EPO increases the number of PV+/WFA– cells during the first two postnatal weeks and accelerates PNN formation at specific early postnatal development windows.

### Increased GABA_A_R cluster density in CA1 pyramidal cells of Tg21 mice at P14

**Figure 4.**
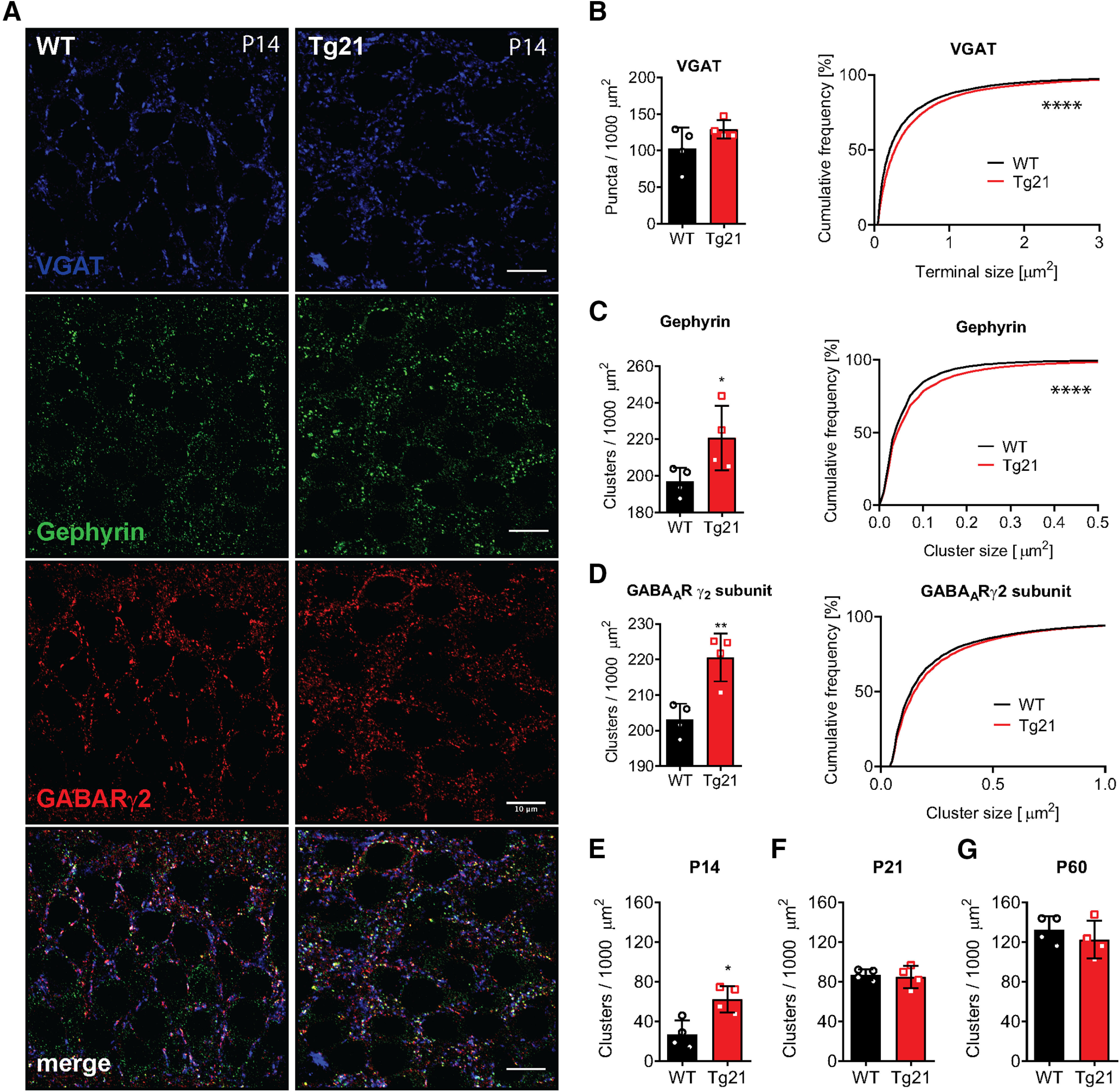
Increased GABA_A_R cluster density in CA1 pyramidal cells of Tg21 mice at P14. ***A***, Representative images of immunofluorescence staining against VGAT: vesicular GABA transporter (blue), gephyrin (green), and γ2-GABA_A_R subunit (red) in WT and Tg21 mice. ***B***, Quantification of presynaptic (VGAT) terminals in WT and Tg21 mice showing increased terminal size (cumulative plot) in Tg21 mice. ***C***, Quantification of postsynaptic clusters of gephyrin showing increased cluster number (bar graph) and size (cumulative plot). ***D***, Quantification of γ_2_-GABA_A_R subunits in WT and Tg21 mice showing increased density (bar graph) in Tg21 mice. Density of postsynaptic (triple labeled) clusters in WT and Tg21 mice at P14 (***E***), P21(***F***), and P60 (***G***). Bar graphs show mean ± SD, data points represent individual mice, *N* = 5 animals per age and genotype, Student’s *t* test (***B–J***). Cumulative frequency plots show total number of quantified clusters in WT and Tg21 animals; *p <* *0.05, **p <* *0.01, ***p <* *0.001, ****p <* *0.0001. Scale bar: 10 μm.

Next, we examined morphologically, whether the density of GABAergic synapses is affected in Tg21 mice. We studied both perisomatic synapses (primarily formed by PV+ basket cells) in the CA1 stratum pyamidale and dendritic synapses in the CA1 stratum radiatum at P14, P21, and P60. Synapses were identified by triple immunofluorescence staining for the VGAT as a marker of presynaptic terminals, the GABA_A_R γ_2_-subunit, which is ubiquitous in GABAergic postsynaptic densities, and the scaffolding protein gephyrin, which closely interacts with GABA_A_Rs (Fig. 4).

Colocalization of these three markers in puncta (clusters) detected by confocal laser scanning microscopy was considered to represent individual GABAergic synapses, which were quantified accordingly. At P14, a 2-fold increase in cluster density was observed in the Tg21 mice as compared with WT (*t* test, *p *=* *0.0105; Fig. 4*E*). At later developmental time points P21 and P60, no differences were detected between genotypes (Fig. 4*F*,*G*). We observed that at P14 all individual markers (Fig. 4*B–D*) were increased in the same proportion (∼20–30%) in Tg21 mice (Fig. 4*B–D*, left column, *C*, *t* test, *p *=* *0.047, *D*, *t* test, *p *=* *0.0050), although VGAT staining did not reach statistical significance (*t* test, *p *=* *0.1507; Fig.4*B*).

**Figure 5.**
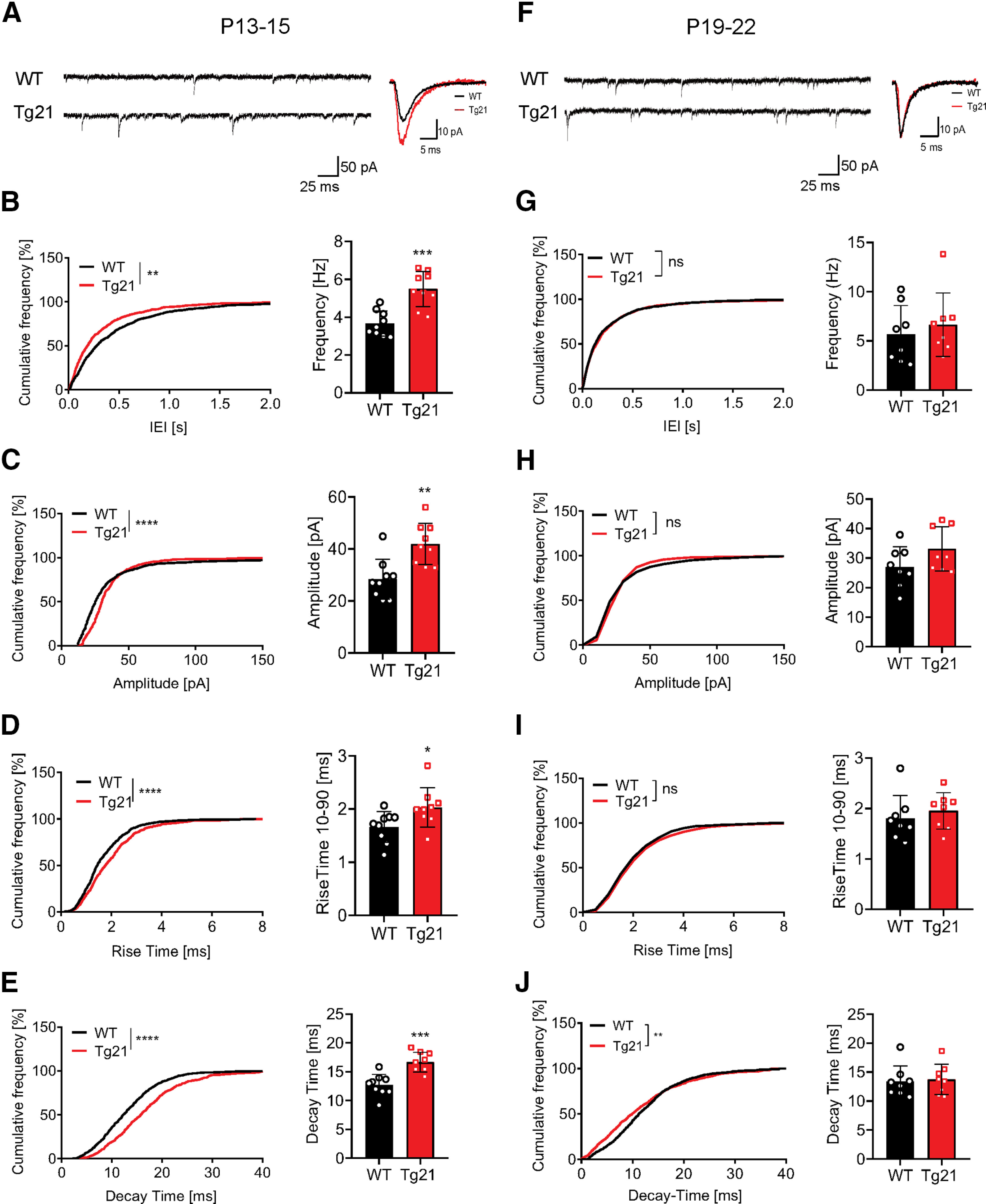
Enhancement of GABAergic synaptic transmission in CA1 area of Tg21 mice at P13–P15. ***A***, Representative raw and averaged mean traces of the mIPSCs in WT and Tg21 mice at P13–P15. ***B–E***, Cumulative frequency distribution plots and data points graphs at P13–P15 of (***B***) IEIs and frequency, (***C***) amplitude, (***D***) rise-time constant, and (***E***) decay-time constant of the mIPSCs from WT (black bars) and Tg21 (red bars). An increase in frequency, amplitude, rise time, and decay time is observed in Tg21mice at P13–P15. ***F***, Representative raw and averaged mean traces of the mIPSCs for WT and Tg21 mice at P19–P22. ***G–J***, Cumulative frequency distribution plots at P19–P22 of (***G***) IEIs and frequency, (***H***) amplitude, (***I***) rise-time constant, and (***J***) decay-time constant. No differences are observed in Tg21 mice at P19–P22. Graphs show mean ± SD, data points represent individual mice, *N* = 9 animals per genotype (P13–P15) and *N* = 8 animals (P19–P22), Student’s *t* test. Cumulative frequency plots show total number of events in WT and Tg21 animals, KS tests; *p <* *0.05, **p <* *0.01, ***p <* *0.001, ****p <* *0.0001.

Assessing the size of VGAT+ puncta, representing presynaptic terminals, and GABA_A_Rγ2/gephyrin postsynaptic clusters, by cumulative frequency analysis (Tyagarajan et al., 2011), we observed a significant increase for all three markers at P14 (KS test, *p *<* *0.0001; Fig. 4*B–D*, right column), suggesting that EPO influences both the size of presynaptic terminals and the aggregation of GABA_A_Rs at postsynaptic sites. These results indicate that EPO increases the formation of GABAergic synapses at the soma and proximal dendrites of CA1 pyramidal cells. Our data corroborates what was previously observed in the formation of PNNs, that EPO accelerates the maturation of the GABAergic system.

We also analyzed EPO’s impact on the density of GABAergic synapses in pyramidal cells and interneuron dendrites in the stratum radiatum of the CA1 area. In contrast to our observations in the stratum pyramidale, no significant difference in GABAergic synaptic clusters was detected (data not shown), suggesting that EPO mainly affects GABAergic synapses from PV interneurons which occur preferentially on the soma and proximal dendrites of pyramidal cells.

### Enhancement of GABAergic synaptic transmission in CA1 area of Tg21 mice at P13–P15

The increase in synaptic GABAergic markers in CA1 pyramidal cells at P14 raised the possibility that this morphologic change has a functional correlate. We measured sIPSCs and action potential-independent miniature (mIPSCs). Since we obtained very similar findings for both populations of IPSCs, only the results from the mIPSC analysis are presented here (Fig. 5).

In pyramidal cells recorded in acute hippocampal slices of WT and Tg21 mice at P13–P15, mIPSC baseline noise of WT and Tg21 animals was in average 2 pA. Tg21 animals had shorter interevent intervals (KS test, *p *<* *0.01; Fig. 5*B*) and hence higher frequency (*t* test, *p *<* *0.001); as well as larger amplitudes (KS test, *p *<* *0.0001, *t* test, *p *<* *0.01; Fig. 5*C*) compared with WT. Additionally, the Tg21 kinetics were slower, with rise-time and decay-time constants being significantly longer than in WT [rise time: KS test, *p *<* *0.0001, *t* test, *p *<* *0.05 (Fig. 5*D*); decay time: KS test, *p *=* *0.0002, *t* test *p *<* *0.001 (Fig. 5*E*)]. Therefore, overall GABAergic transmission onto pyramidal cells of Tg21 mice is enhanced. The larger amplitude of mIPSC correlates well with the increased size of GABA_A_R clusters and suggests an increase in synaptic strength.

In slices taken from P19–P22 mice, no differences in interevent intervals/frequency (Fig. 5*G*) or event amplitude (Fig. 5*H*) of GABAergic mIPSCs were observed between Tg21 and WT mice. These results are in line with the morphologic analysis of GABAergic synaptic density at P21, in which no differences in synaptic clusters were observed between genotypes. At this age, mIPSCs kinetics in Tg21 mice became faster and equal to WT mice (Fig. 5*I*,*J*).

### EPORs are highly expressed in Tg21 mice CA1 pyramidal cells during early postnatal development

**Figure 6.**
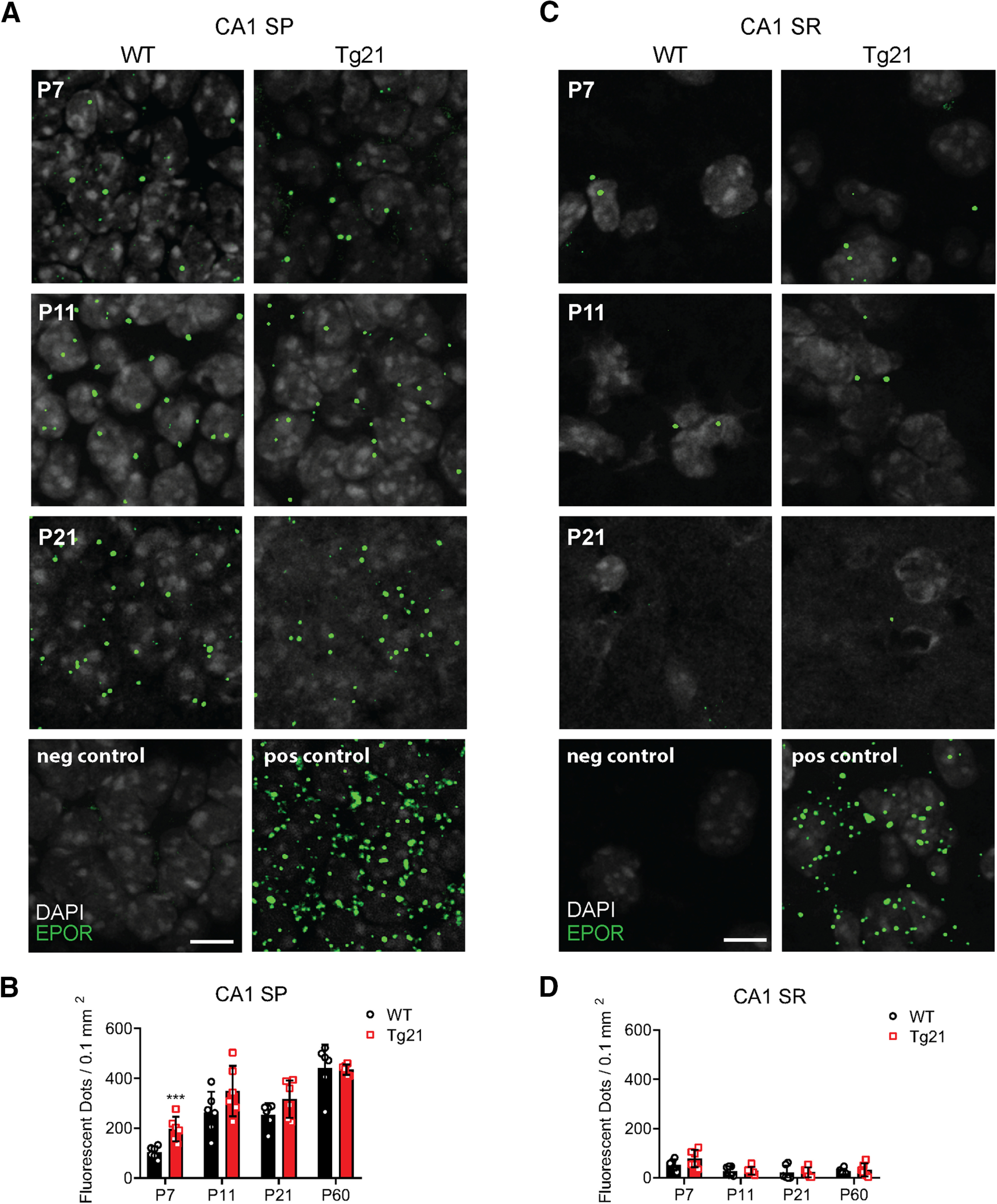
EPORs expression in the CA1 area is restricted to principal cells. Representative images of fISH for mRNA EPORs (green dots) colabeled with DAPI (gray) in WT and Tg21 mice at P7, P11, and P21 in (***A***) stratum pyramidale (SP) and (***C***) stratum radiatum (SR). Negative control (P7): fluorophore; positive control (P7): housekeeping gene. Scale bar: 10 μm. Quantification of EPOR mRNA dots in CA1 SP (***B***) and CA1 SR (***D***) at different postnatal ages, showing its selective presence in SP throughout postnatal development. More EPOR mRNA dots are quantified at P7 in Tg21 mice. Data are given as mean with individual values ± SD, *N* = 6 animals per genotype and age. Two-way ANOVA test; CA1 SP, ***p <* *0.001.

We next evaluated the cellular expression of EPORs in the hippocampus from WT and Tg21 mice. To this end, we performed fISH (RNA scope) of EPOR mRNA at postnatal ages P7, P11, P21, and P60. In both genotypes, EPOR expression was found in pyramidal cells of the CA1 area (Fig. 6*A*,*B*) and in a reduced number also in cells in the stratum radiatum (Fig. 6*C*,*D*). This finding strongly suggests a selective expression of EPOR mRNA in pyramidal cells throughout postnatal development. Quantification of EPOR mRNA probes in the stratum pyramidale of the CA1 area showed a significant age effect with increasing density of mRNA puncta until P60. Between P7 and P11, EPOR mRNA expression was mostly increased, reaching values like P21 and not significantly different from P60. At P7, Tg21 mice showed a 2-fold higher EPOR mRNA expression. No change in EPOR between genotypes was observed at any other age.

### Constitutive deletion of EPORs from Gad65 cells has no impact on the GABAergic system

**Figure 7.**
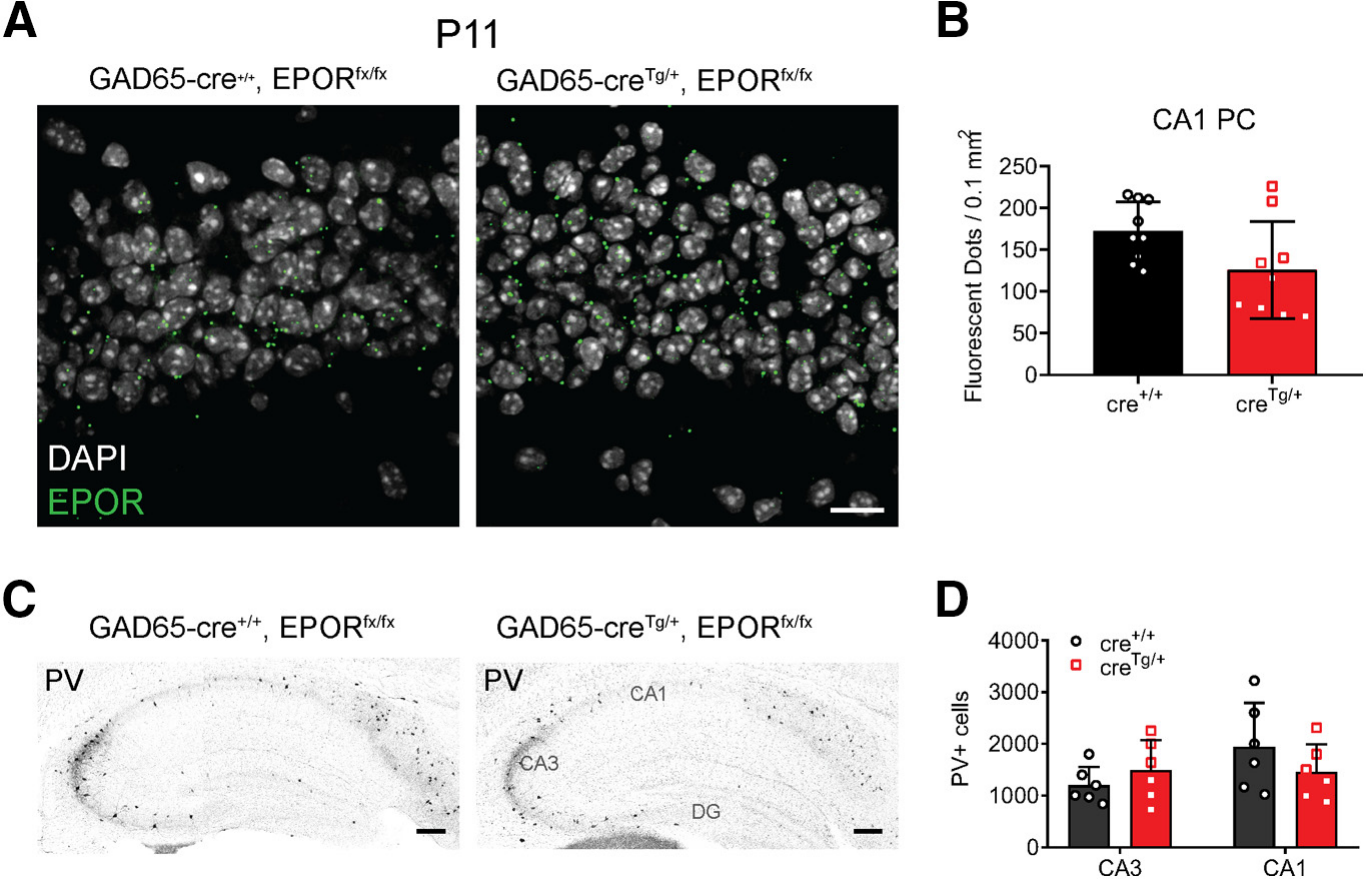
Constitutive deletion of EPORs from Gad65 cells has no impact on the GABAergic system. ***A***, Representative images of fISH for EPORs in GAD65-cre^+/+^, EPOR^fx/fx^ mice and GAD65-creT^g/+^, EPOR^fx/fx^ (GAD65: Glutamat decarboxylase isoform 65) mice at P11 in CA1 stratum pyramidale area. ***B***, Quantification of EPOR mRNA dots in CA1 area, showing no effect of the targeted mutation. ***C***, Representative images of PV immunoperoxidase staining in GAD65-cre^+/+^, EPOR^fx/fx^ mice and GAD65-creT^g/+^, EPOR^fx/fx^ mice at P11 showing no change in PV immunostaining. ***D***, Unbiased quantification of PV^+^ cell numbers in CA1 area shows no alteration in cell numbers on deletion of EPOR in interneurons. Data are given as mean ± SD, *N* = 3 animals and 3 hippocampi per genotype for fISH and *N* = 6 animals per genotype for PV stereology. Two-way ANOVA test. Scale bars: 10 μm (***A***) and 200 μm (***C***).

To further ensure that EPORs are mainly expressed in principal cells, fISH of EPOR mRNA along with immunolabeling of PV+ cells was performed but no EPORs were found on PV+ cell in the hippocampus. Furthermore, the lack of EPOR expression on GABAergic cells was confirmed with the conditional deletion of EPOR (EPOR^fx/fx^) in the GAD65-Cre^Tg/+^, EPOR^fx/fx^ mouse line. In this line, EPOR expression was measured by fISH analysis at P11 (age when EPORs are highly incremented) in CA1 stratum pyramidale. Similar numbers of labeled puncta were detected in GAD65-Cre^Tg/+^, EPOR^fx/fx^ and control GAD65-Cre^+/+^, EPOR^fx/fx^ (unpaired *t* test, *p *=* *0.063; Fig. 7*A*,*B*). Also, the number of PV+ cells in hippocampus were quantified at P11, showing no differences between control and gene-targeted mice (two-way ANOVA, *F*_(1,12)_ = 0.036, *p *=* *0.8523; Fig. 7*C*,*D*), nor in neuropil formation, suggesting that the effects of EPO on the GABAergic system are most likely independent of EPOR expression in interneurons.

### Increased density of glutamatergic terminals on PV+ interneurons of Tg21 mice at P14

**Figure 8.**
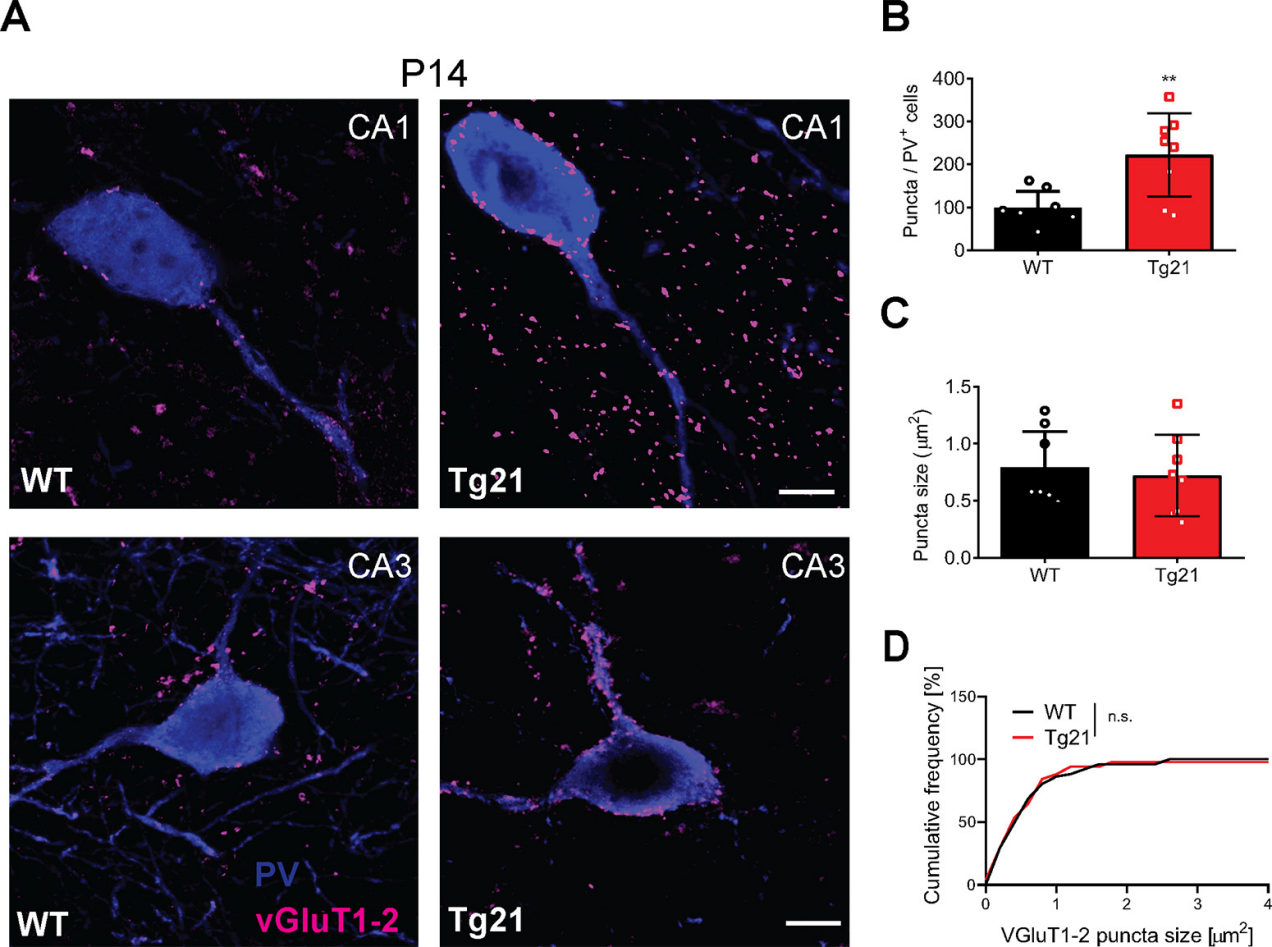
Increased density of glutamatergic terminals on PV+ interneurons of Tg21 mice at P14. ***A***, Representative images of double immunofluorescence staining for VGluT1-2 (magenta) and PV (blue) in hippocampus of WT and Tg21 mice. ***B***, Quantification of VGluT1&2+ puncta in PV+ cells, showing higher numbers in Tg21 mice. ***C***, ***D***, No change in puncta size between genotypes was observed. Bar graphs are given as mean ± SD, *N* = 4 animals per genotype and 2 hippocampi per animal. 10 to 15 PV+ cells were quantified per hippocampi and area. Student’s t test, **p, 0.01, KS tests n.s. Scale bar: 10 mm.

Selective EPOR mRNA expression in principal cells of WT and Tg21 mice suggests that EPO might exert a trophic effect on pyramidal cells, which are known to strongly innervate neighboring PV+ cells (feed-forward excitation). Therefore, we tested the hypothesis that glutamatergic input might be increased in PV+ interneurons in Tg21 mice. To this end, we used double immunofluorescence for PV and the two VGluT1 and VGluT2 in the CA1 and CA3 area at P14, the postnatal age where most changes in GABAergic transmission were observed (Fig. 8). Overall density of VGluT1/2-immunoreactive puncta revealed a significantly higher density of VGluT1/2-immunoreactive terminals contacting PV+ interneurons in CA1 and CA3 (unpaired *t* test, *p *=* *0.0011; Fig. 8*A*,*B*), with no change in the size of these terminals (Fig. 8*C*,*D*), suggesting that increased synaptic excitatory inputs onto PV cells might drive the accelerated maturation of PV+ cells observed in Tg21 mice.

Thus, in summary, we conclude that the effect of EPO on GABAergic postnatal maturation results from pyramidal regulation of interneuron network formation and survival.

## Discussion

The present study shows that constitutive neuronal overexpression of EPO stimulates maturation of the GABAergic system in the mouse hippocampus. EPO overexpression affected numerous aspects of GABAergic maturation during the second and third postnatal window, namely elevated expression of GABAergic cells and markers of GABAergic neurons (mainly PV, SST, and NPY), increased GABAergic synapse density and function in pyramidal cells, faster maturation of PV+/WFA+ cells, and increased innervation of PV+ interneurons by glutamatergic terminals. All these changes suggest enhanced GABAergic function during a critical period for the proper formation of brain circuits. EPO overexpression in neurons caused a reduction in apoptosis in the CA1 and CA3 hippocampus during the first postnatal week, when EPORs were upregulated in principal cells of the Tg21 mice. Therefore, survival of interneurons during this postnatal window is essential for postnatal development and circuit formation. The lack of EPORs on interneurons, confirmed by the lack of a phenotype observed in the Gad65-cre, EPOR^fx/fx^ mice, additionally suggests pyramidal cells to regulate interneuron survival (Wong et al., 2018). Our results support the notion that EPO can indirectly stimulate the development of GABAergic transmission, which in turn is a key driver of neural circuit formation. Therefore, EPO could be highly beneficial in pathologic conditions that affect GABAergic neurons and might protect the brain against an imbalance of excitatory/inhibitory transmission.

We showed an increase in GABAergic neurons (mainly PV, SST, and NPY) during postnatal hippocampal development caused by a decrease in apoptosis during the first postnatal week. The reduction in apoptosis was mainly observed in the stratum pyramidale of CA3 and CA1 areas and in the CA1 stratum oriens, which compress oriens-lacunosum moleculare interneurons (O-LM) that express SST and NPY. The restricted location of cell somata in the CA1 stratum oriens dictates that the source of excitatory inputs is mainly from pyramidal cells, therefore survival and enhancement of glutamatergic inputs is also expected to occur in SST and NPY cells. At P14 PV-expressing cells are increased mainly in the CA3 area, whereas SST-expressing cells were increased in the CA1 area. Most cells in the CA1 stratum oriens showed reduced apoptosis at P7, therefore the increase in SST number could be linked to survival.

Whether EPO-mediated enhancement of interneurons is also linked to EPO signaling on inhibitory neurons during embryonic origin is unlikely, since deletion of EPORs on Gad65-cre cells had no effect on normal GABAergic development, and EPORs start to express postnatally in the hippocampus. In the neocortex, EPO and EPORs are expressed embryonically in the neurogenic ventricular zone and EPO signaling is required for radial migration and laminar positioning of upper-layer excitatory neurons (Constanthin et al., 2020). In contrast to the cortex, we show in this study that EPO signals in the hippocampus postnatally. We show that EPORs are expressed on CA1 pyramidal neurons and the expression increases postnatally to reach a zenith at early adulthood (P60). It is important to emphasize that in we have confirmed activation of the ERK and AKT pathway across postnatal development in the hippocampus of Tg21 mice and in a model of stroke (Kilic et al., 2005). Therefore, EPO-mediated activation of PI3K-AKT and Erk1/2 extends from neuroprotection to influencing neuronal differentiation postnatally. Activation of AKT signaling is known to mediate phosphorylation of the GABA_A_R γ_2_ subunit, resulting in increased GABA_A_R trafficking to the postsynaptic membrane and facilitation of GABAergic inhibitory transmission (Wang et al., 2003). Furthermore, clustering of gephyrin at postsynaptic sites is modulated by various posttranslational modifications, notably by PI3K/Akt (Tyagarajan and Fritschy, 2014). Modulation of gephyrin phosphorylation has been shown to influence the density of GABAergic synapses on dendrites *in vitro* and *in vivo* (Tyagarajan et al., 2011). In our model, we observed an increase in GABA_A_Rγ_2_ postsynaptic clusters and an increase in GABA_A_R γ_2_ and gephyrin cluster size, along with enhanced IPSCs during the postnatal development. Since EPORs are highly expressed in CA1 pyramidal cells starting from P11, we propose that EPO signaling onto pyramidal cells activates the PI3K/Akt pathway, leading to an increase in GABA_A_R trafficking to the membrane and resulting in increased synaptic GABAergic function in CA1 pyramidal cells. ERK1/2 phosphorylation is also implicated in the differentiation of neurons (Li et al., 2006) and synaptic hippocampal plasticity in the CA1 area (Kanterewicz et al., 2000). However, no evidence for *in vivo* ERK1/2 regulation of GABA_A_Rs has been reported so far. Furthermore, EPO causes an increase in presynaptic VGAT size. Therefore, the effect of EPO overexpression on the GABAergic synapses is likely presynaptic and postsynaptic, through the bidirectional signaling of the neuroligin-neurexin complex (Shen and Scheiffele, 2010). Additionally, it is possible that EPO acts via activation of the TrkB/BDNF pathway, causing an increase in presynaptic VGAT expression (Wang and Xia, 2015).

We show, by the evaluation of PNN formation, that EPO overexpression does not change the onset nor the number of PV+ cells surrounded by PNNs but enhances PV and PNN staining intensity. This suggests that EPO accelerates interneuron maturation. The increase in maturation is in line with the observed increase of excitatory VGluT1 and VGluT2 inputs to PV+ cells and the strengthening of inhibitory synapses to pyramidal neurons in the CA1 area, as seen at P13–P15 by the increase in mIPSC frequency and amplitude. Interestingly, in Tg21 mice, PV+/WFA+ cells exhibit significantly larger soma at P11, leading to the question of whether PV+ cell size is also related to increased synaptic plasticity. PV expression is linked to synaptic density ratios. Specifically; high PV expression means more differentiation and a higher excitatory/inhibitory ratio (Donato et al., 2013). Additionally, PNNs preferentially form around more mature PV+ cells with a higher staining intensity (Donato et al., 2013). No changes in PV staining intensity and soma size were seen at other postnatal ages, and at P26 the number of PV+ cells surrounded by PNNs was equal in both genotypes. Therefore, EPO may stimulate the maturation of late PV-basket cells, which exhibit plasticity when the critical period of plasticity is induced (Donato et al., 2015). Indeed, an increase in PV+/WFA– cells, in which plasticity remains high, was quantified in Tg21 mice at P11.

PV+ basket cells provide powerful perisomatic feedforward and feedback inhibitory inputs onto CA1 pyramidal cells (Klausberger and Somogyi, 2008). The maturation stage of PV-basket cells has been implicated in different forms of learning since each interneuron type selectively gates distinct information flow to pyramidal cells. Accordingly, changes at different times within the period of plasticity might differ in network formation and the response to specific learning requirements. The capability of EPO to increase, on the one hand, the number of immature PV+ cells and to stimulate, on the other hand, the maturation of PV-basket cells makes this paradigm relevant for therapeutic applications in the treatment of developmental disabilities.

One of the central interests in neurodevelopmental therapy is to identify pharmacological interventions that stimulate neural synaptic plasticity. Moreover, the hippocampus is an area of high clinical interest, since the integrity of synaptic function is implicated in several disease states caused by perinatal injury (Travaglia et al., 2016; Alberini and Travaglia, 2017), and early interventions are required. Many studies reported EPO as an efficient neuroprotective agent in models of experimental stroke (Wiessner et al., 2001), cerebral hypoxic-ischemia, PBI, and neuroinflammation (Sirén et al., 2009). In the hippocampus, it inhibits apoptosis associated with glutamate toxicity, and promotes survival and neurogenesis (Doggrell, 2004; Mennini et al., 2006; Hassouna et al., 2016; Zhang et al., 2018). Several clinical trials in neonatology using a high dose of recombinant human EPO given in the first days of life (from 500 to 5000 IU/kg, i.p., to pass the blood brain barrier) have shown that EPO cannot only reduce acute injury but represents a promising tool for long-lasting prevention of trauma-induced developmental delay, as well as cognitive and neurobehavioral dysfunction (Sirén et al., 2009; Natalucci et al., 2016). Accumulating evidence from animal studies suggests EPO is an enhancer of hippocampal synaptic plasticity and cognition in the mature hippocampus (Adamcio et al., 2008; Dias et al., 2018), as well as in patients with psychiatric diseases (Ehrenreich et al., 2007a,b, 2008; Miskowiak et al., 2010, 2015, 2016). However, whether EPO can stimulate neurodevelopment and specifically the maturation of the inhibitory synaptic transmission has remained elusive. With this work, we provide an ideal model to amplify EPO’s physiological effects during postnatal brain development without altering blood cell production. We could show EPO’s potential in stimulating postnatal maturation of neurons in the hippocampus, and additionally, we identified the targets through which EPO signals. The impact of EPO on GABAergic interneurons is a network effect in which increased glutamatergic inputs to PV+ cells and increase GABAergic inputs to pyramidal cells is observed early in postnatal development.

Our work supports the use of EPO to stimulate neurodevelopment. Since EPO is controlled via the HIF-2/prolyl hydroxylase 2 (PHD2) pathway (Takeda et al., 2008; Gassmann and Muckenthaler, 2015), inhibitors of PHD2 to stabilize the α-subunit of HIF-2 and subsequently increase transcription of the EPO gene are being commercialized (Soni, 2014). These components are small and can cross the blood brain barrier, so it is to be expected that they will inhibit PHD2 activity in neurons, thus being a potential therapy to increase neuronal EPO production and stimulate neuronal maturation.

In summary, our data provide evidence that EPO accelerates the maturation of the GABAergic system in the neonatal (P7–P14) hippocampus, without causing network imbalance. The results support the use of EPO as a therapeutic agent to stimulate normal brain development after PBI.

## Synthesis

Reviewing Editor: Susanne Wolf, Charite University Hospital Decisions are customarily a result of the Reviewing Editor and the peer reviewers coming together and discussing their recommendations until a consensus is reached. When revisions are invited, a fact-based synthesis statement explaining their decision and outlining what is needed to prepare a revision will be listed below. Note: If this manuscript was transferred from JNeurosci and a decision was made to accept the manuscript without peer review, a brief statement to this effect will instead be what is listed below.

This paper identifies a modest, transient effect of erythropoietin receptor activation on interneurons. The study is well-executed and the authors addressed all comments of the reviewers carefully. The revised version has thus improved and is accepable for publication in eNeuro. The study fulfills eNeuro's aim to publish scientifically sound studies that present new observations that may not yet have mechanistic underpinnings.
